# Case Report: Next-Generation Sequencing Reveals Tumor Origin in a Female Patient With Brain Metastases

**DOI:** 10.3389/fonc.2021.569429

**Published:** 2021-04-12

**Authors:** Qun Li, Xiaoyan Zhang, Jiao Feng, Dezhi Cheng, Lin Cai, Zhang’an Dai, Shuyu Zhao, Jianmin Li, Jingjing Huang, Yu Fang, Honglin Zhu, Danhua Wang, Sizhen Wang, Tonghui Ma, Xianghe Lu

**Affiliations:** ^1^ Neurosurgery department, The First Affiliated Hospital of Wenzhou Medical University, Wenzhou, China; ^2^ Department of Translational Medicine, Genetron Health (Beijing) Technology, Co. Ltd., Beijing, China; ^3^ Holistic Integrative Pharmacy Institutes and Comprehensive Cancer Diagnosis and Treatment Center, College of Medicine, Hangzhou Normal University, Hangzhou, China; ^4^ Thoracic surgery department, The First Affiliated Hospital of Wenzhou Medical University, Wenzhou, China; ^5^ Pathology department, The First Affiliated Hospital of Wenzhou Medical University, Wenzhou, China; ^6^ Genetron Health (Beijing) Technology, Co. Ltd., Beijing, China

**Keywords:** next-generation sequencing, undifferential thyroid cancer, *TERT* promoter mutation, *RET* fusion, anlotinib, tumor origin

## Abstract

**Background:**

Brain metastasis mainly originates from lung cancer. Napsin A and TTF-1 factors have frequently been detected in lung adenocarcinoma cases. Brain metastasis tumors with napsin A and TTF-1 positive are easily classified as lung adenocarcinoma origin. However, some thyroid cancers also exhibit these clinical features. Besides, lung is the most common metastasis of undifferential thyroid cancer. Therefore, it requires development of novel diagnostic tools to aid in distinguishing between pulmonary and thyroid origin.

**Patient Findings:**

We reported a case that was initially diagnosed as brain metastatic lung cancer based on immunohistochemistry results. Analysis of next-generation sequencing (NGS) data from the brain lesion revealed that the cancer may have originated from the thyroid. We detected combo mutations in *TERT* promoter mutation, *RET* fusion and *TP53*, which are common in undifferential thyroid cancer (UTC), but rare for lung cancer. These results, coupled with identification of PAX8, indicated that this patient had UTC. Additionally, her three sons, despite being asymptomatic, were all diagnosed with papillary thyroid carcinoma.

**Summary:**

The patient received anlotinib treatment and showed good clinical outcomes. One month after anlotinib treatment, the pulmonary nodules were found to be controlled, and the thyroid tumor drastically reduced, and tracheal compression relieved. She continued anlotinib treatment for the following two months, but died one month later because the treatment stopped owing to financial reasons. All her sons underwent total thyroidectomy with lymph node dissection.

**Conclusions:**

Although NGS has been reported to assist in diagnosis of the origin of some tumors, this is the first evidence of NGS for the determination of the origin of thyroid tumors. To our knowledge, this is the first time that a combination of multiple mutations has been used to help determine the origin of a tumor, compared with the previous single mutant gene. Moreover, this is the first evidence on the use of anlotinib for treatment of UTC with distant metastasis. Besides, all three sons of the patient had thyroid carcinoma in subsequent examinations, indicating high-risk for familial non-medullary thyroid cancer in UTC patients and necessity for performing thyroid ultrasound testing in other family members.

## Introduction

Brain metastasis results from various types of cancer, key among them being lung cancer (1). Clinical diagnosis of lung adenocarcinoma results from positive expression of napsin A aspartic peptidase (napsin A), and transcription termination factor-1 (TTF-1) markers (2, 3). In fact, brain metastasis tumors that positively express napsin A and TTF-1 are always classified as lung adenocarcinoma origin.

Undifferential thyroid cancer (UTC) is the least common type accounting for 1-2% of thyroid cancer cases. In fact, advanced distant metastatic disease is the most challenging condition among patients (4, 5). Intrathoracic, neck lymph nodes and lungs are the most common metastatic sites of UTC (6, 7). UTC often expresses PAX8 and loses thyroid cancer-specific marker-thyroglobulin (TG), and some of UTC tumors show positive napsin A and TTF-1 expression (3, 8). Immunophenotyping of the metastases for some UTC tumors is, therefore, similar to that for primary lung adenocarcinoma. Next-generation sequencing (NGS) has been successfully applied in clinical diagnosis, enabling identification of genetic features. Oncogenetic mutations in the MAPK (*BRAF*, *RAS*), PI3K and mismatch repair pathways, *RAC1*, *TP53*, and *TERT* promoters, as well as *CCDC6-RET* fusion have often been identified in UTC. However, mutations in the *TERT* promoter and *RET* fusion have been rarely detected in lung cancer (9, 10). Despite identification of several oncogenic mutations, only one mutation-driven targeted therapy has so far been approved for *BRAF*-mutant UTC (10).

Anlotinib is an oral novel multi-target tyrosine kinase inhibitor that targets RET, vascular endothelial growth factor receptor (VEGFR), fibroblast growth factor receptor (FGFR), platelet-derived growth factor receptors (PGFR) and c-kit (11, 12). When compared to sunitinib, anlotinib exhibits significantly lower side effects. Consequently, anlotinib has currently become an effective compound for inhibition of multi-targeting receptor tyrosine kinases, owing to its efficacy against multiple cancers, including lung cancer (13–16), advanced medullary thyroid cancer (17), soft tissue sarcoma and metastatic renal cell carcinoma (12). However, its potential to treat UTC has not been reported.

In the current study, we studied a case that was initially considered brain metastatic lung cancer based on the immunohistochemistry (IHC) results, but found to be cancer originating from thyroid after NGS of the brain lesions. Specifically, we identified combo mutations in, *TERT* promoter, *RET* fusion and TP53, which are common for UTC but rare for lung cancer. We administered anlotinib treatment to the patient and achieved good clinical outcomes. This is a first study reporting the use of anlotinib for UTC with lung metastasis.

## Case description

**Figure 1:**
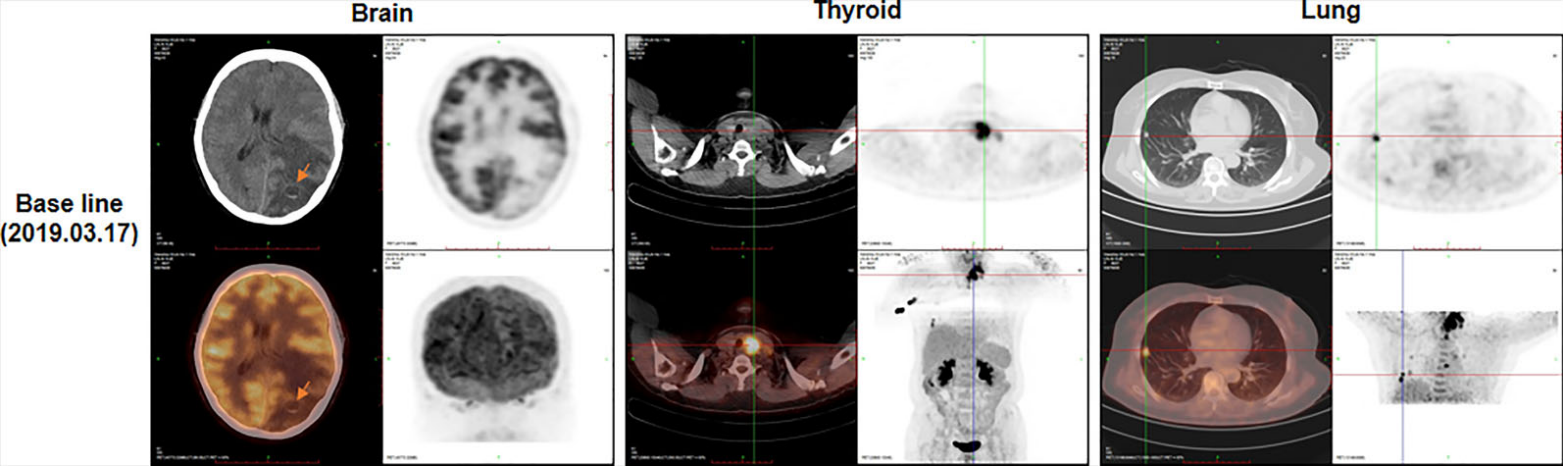
The positron emission tomography-computed tomography (PET-CT) at baseline (before surgery). PET-CT showed masses in brain (arrow), thyroid and lung.

**Figure 2:**
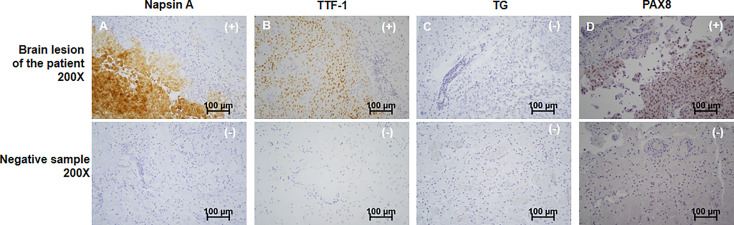
Immunohistochemistry of the patient’s brain cancer. From top to bottom, the immunochemistry results of the patient’s brain tumor and negative control sample were shown. The patient’s tumor showed positive of Napsin A **(A)**, TTF-1 **(B)** and PAX8 **(D)**, showing as “(+)” in top right corner of the graphs. Negative of TG **(C)** was identified, presenting as “(-)” in top right corner of the graphs. Corresponding negative results from negative control sample were also shown. Scale bar was in the lower right corner of each image. All images were magnified by 200 times. Scale bars (100μm) were provided.

**Figure 3:**
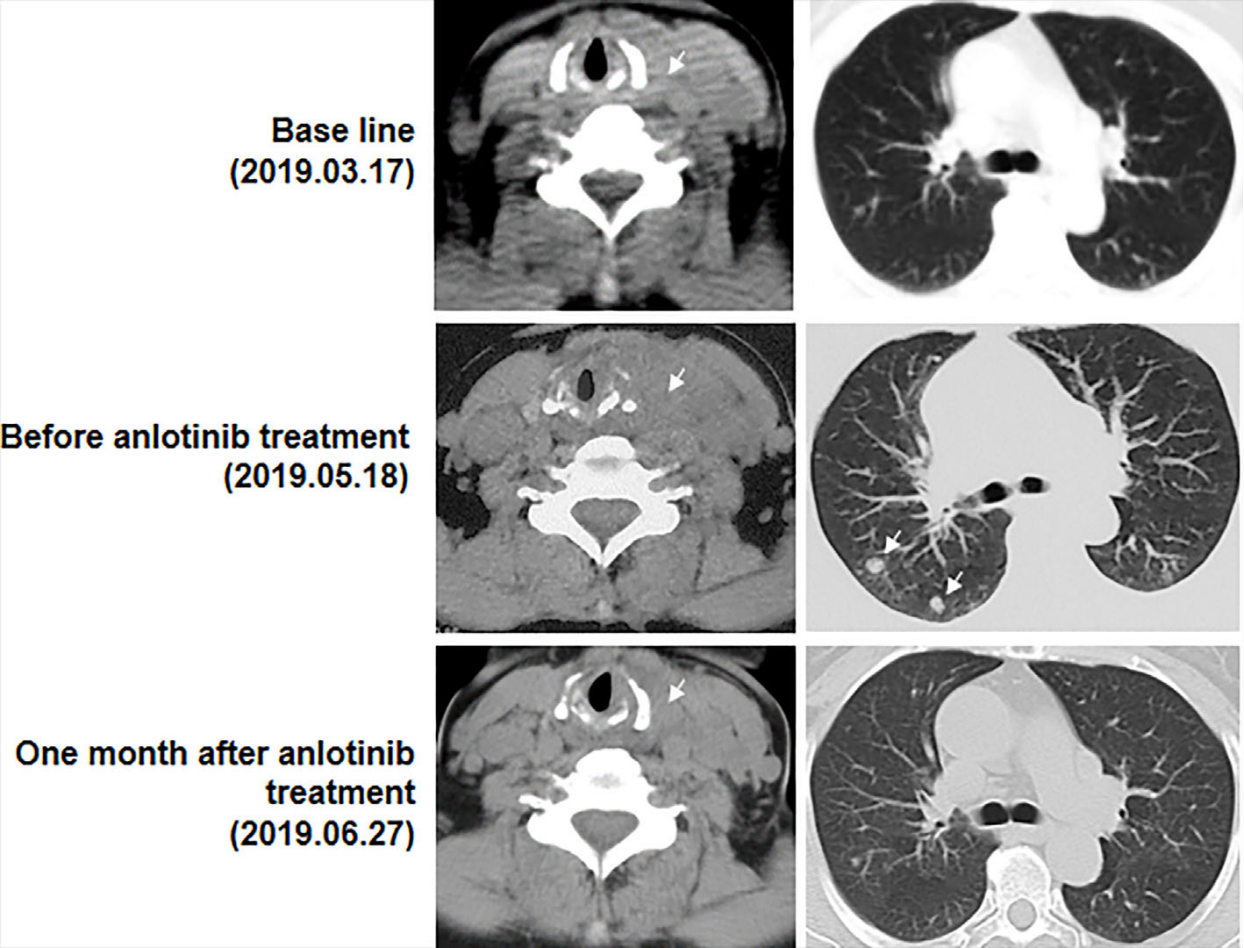
Computed tomography images (CT) of the thyroid and lung before and after anlotinib treatment. Arrows represent the masses. At base line, in addition to brain lesions, there were a mass in the left thyroid and some opaque mottled shadows and pulmonary nodules in the lung. Then the patient experienced brain surgery. Before anlotinib treatment, a huge mass in the left thyroid and two obvious pulmonary nodules was found. One month after anlotinib treatment, the pulmonary nodules were found to be controlled, thyroid tumor drastically reduced and tracheal compression relieved.

A 63-year-old female patient was presented to hospital, in March 2019, with repeated dizziness and visual impairment. A computerized tomography/magnetic resonance imaging (CT/MRI) revealed a mass lesion in the left occipital lobe. In addition to the brain mass, positron emission tomography (PET)-CT also indicated a hyper-metabolic mass in the left thyroid as well as some opaque mottled shadows and pulmonary nodules in the lung (**Figure 1**). Further ultrasound revealed multiple thyroid nodules, the largest calcified one located in the left thyroid and measuring 41*30*44 mm. The patient was subjected to brain tumor resection. IHC studies of the resected brain tumor positively identified cytokeratin 7 (CK7), napsin A and TTF-1, but TG was negative (**Figures 2A–C**; Details on IHC methods were showed in **Supplementary Data**). Fine-needle aspiration (FNA) of the thyroid lesion identified clusters of undifferentiated tumor cells. Based on these results, the pathologist hypothesized that the lesions in brain and thyroid had migrated from undifferentiated lung adenocarcinoma. However, the NGS results from FSZ-Thyroid NGS Panel V1 (Genetronhealth) revealed *TERT* promoter mutation, *RET* fusion and *TP53* mutation in the brain lesion (**Table 1** and **Supplementary Figure 1**). The details of NGS were shown in the **Supplementary Data**. These mutations combo are indicative of undifferential thyroid cancer (9), and rarely occur in lung cancers. Since the patient did not consent to further surgery, we did not obtain any operation samples on the thyroid or lung for further analysis. In addition, we positively detected PAX8 through IHC (**Figure 2D**). This factor is negative in lung adenocarcinoma, but always positive in thyroid cancer (18). Based on these results (positive PAX8, negative TG, cytopathology of thyroid FNA and the special genomic features), we concluded that the patient had undifferential thyroid cancer, with lung and brain metastasis. Due to the patient with brain metastasis and without *BRAF* mutations, RET-targeted BLU-667 clinical trial and BRAF inhibitor were not suitable for this patient. Therefore, indication therapy or off-label treatment were needed for this patient. After comprehensive consideration, we administered anlotinib, a novel multi-kinase inhibitor that has also been found to block RET (11). One month after treatment, the pulmonary nodules were found to be controlled, thyroid tumor drastically reduced and tracheal compression relieved (**Figure 3**). Unfortunately, the patient stopped anlotinib treatment, after three months, owing to financial constraints. Only two weeks after discontinuation of anlotinib, the patient went back to the hospital exhibiting breathlessness and cough. A CT scan revealed a large mass outbreak in her lungs, and she eventually died of respiratory failure.

**Table 1:** NGS Gene Mutation Profiling of tumor tissues.

Samples	Gene	Mutation type	DNA _ Change	Protein _ Change
Brain tumor of the patient	*TERT*	Upstream promoter mutation	C228T	
	*RET*	Gene fusion	*CCDC6{NM_005436.4}:r.1_303+1_RET{NM_020975.4}:r.2137_5617*	
	*TP53*	Missense mutation	*c.842A>T*	p. D281V
The eldest son	*BRAF*	Missense mutation	*c.1799T>A*	p. V600E
The second son	*BRAF*	Missense mutation	*c.1799T>A*	p. V600E
The youngest son	*BRAF*	Missense mutation	*c.1799T>A*	p. V600E

NGS, next-generation sequencing.

Given the patient’s critical condition, three sons of the proband, who were asymptomatic, were presented to the local hospital and subjected to thyroid ultrasound examinations. They were all found with thyroid lesions. Total thyroidectomy, with lymph node dissection, was performed on all of them. Results revealed a thyroid papillary carcinoma (1.2cm), in the right thyroid lobe and isthmus (without lymph node metastasis) of the 36 years old youngest son. Pathologic diagnosis in the elder son (40 years old) revealed a follicular adenoma in the left lobe as well as a thyroid papillary microcarcinoma in the right lobe and isthmus. Lymph node metastasis were found in Level VI of the neck. Unfortunately, a more extensive lymph node metastasis was further detected at Level II, III, IV and VI of the neck in the eldest son (42 years old). All tumor tissues from the three sons were positive of TTF-1, TG and PAX8, negative of Napsin A (**Supplementary Figure 2**). Targeted sequencing by using FSZ-Thyroid NGS Panel V1 (Genetronhealth) was performed on the tumor tissues of the three sons, the results of which showed all the tissues had BRAF V600E mutations (**Table 1** and **Supplementary Figure 3**).

## Discussion

We present a case of UTC, with lung and brain metastasis, in which genetic features including *CCDC6-RET* fusion, *TERT* promoter and *TP53* mutations were identified. Based on these features, a multi-targeted reagent, anlotinib, was used to treat the patient and resulted in good clinical outcomes. This is the first study reporting the use of mutation combo and IHC for the confirmation of UTC and the use of anlotinib for the treatment of UTC. The most noteworthy point in the present case is that traditional IHC markers combined with genetic characteristic of tumor are more effective in accurately determining the source of metastatic tumor. Generally, IHC is a major diagnostic tool for determining the origin of metastatic tumors (19, 20). However, this diagnosis is not enough for some special cases. A combined detection of napsin A and TTF-1 through IHC has been considered a reliable procedure for diagnosing adenocarcinoma originating from the lungs (2, 3). However, several studies have recently shown that both of Napsin A and TTF-1 are positive in approximately 15% of UTC cases (8). TG, a specific thyroid cancer marker due to its especial expression in tissues of thyroid origin has been reported (3), but its expression is often lost following dedifferentiation of UTC (8). Therefore, a positive napsin A and TTF-1 detection, as well as negative TG, is considered a misdiagnosis for lung adenocarcinoma. Studies have reported that some genetic alterations can be used to aid in determining the source of metastatic carcinoma, with higher specificity than traditional IHC markers (16). For example, *IDH* has been found to be mutated in low-grade gliomas, chondrosarcomas, hematologic malignancies and bile duct cancer (21, 22). *BRCA1* and *BRCA2* are prone to mutations in breast and ovarian cancers, with evidence of *BRCA* gene mutations indicating the genetic origin of these cancers (23). In addition, *TERT* promoter mutations are frequently identified in melanoma, thyroid cancer, bladder cancer and glioblastoma, while they are rare (2.57%) in lung cancer. On the contrary, EGFR L858R mutation is only found in lung cancer (24). Changes in *RET* expression tend to frequently occur in thyroid, and invasive breast cancers, as well as pancreatic ductal adenocarcinomas, but are rare in lung cancer. Moreover, the most common *RET* fusions are *CCDC6-RET* in thyroid cancer and *KIF5B-RET* in lung cancer (25). A combination of mutations including *CCDC6-RET* fusion, *TERT*-promoter and *TP5*3 often occurs in dedifferentiated thyroid cancer (9). However, there is a probability of only 0.005% of this happening in lung adenocarcinoma (24, 25). Combo mutations from NGS in this case clearly confirmed the origin of the thyroid tumors; thus, this is the first case that the determination of the tumor origin by the assist of combo mutations, compared with previous single mutant gene. Moreover, IHC analysis positively detected PAX8, which has been strongly associated with thyroid origin (8), suggesting that this factor is an additional tool for discriminating thyroid from lung cancer in the setting of a double positive of napsin A and TTF-1.

Our results showed that anlotinib is effective in treating UTC, a rare thyroid cancer with a high mortality rate. Conventional treatments, such as surgery, radiotherapy and chemotherapy, have not effectively managed. In fact, the only approved targeted therapy, dabrafenib with trametinib, only works in BRAF^V600E^-positive anaplastic thyroid carcinoma patients. Several novel biological agents, as well as immune checkpoint and aurora kinase inhibitors, are currently under testing for treatment of UTC (26). Case in the current study, a patient with *RET* fusion, who was excluded from RET-targeted BLU-667 clinical trial because of brain metastasis, and the patient without *BRAF* mutation, also excluded from BRAF inhibitor, finally benefited from anlotinib. Anlotinib recently showed durable antitumor activity in lung cancers (13–16), medullary thyroid carcinoma (MTC) (17) and some other cancers (12). To the best of our knowledge, this was the first time that anlotinib has been used to manage UTC. The treatment resulted in good control of primary and lung metastasis lesions. Manageable adverse events were demonstrated in anlotinib-treated lung cancer (15) and MTC (17), here also no serious side effects occurred. These clinical results indicate that anlotinib may develop a new avenue for UTC therapy.

In addition, the patient’s sons all diagnosed with thyroid carcinoma, indicating obvious characteristics of familial non-medullary thyroid cancer (FNMTC). Previous studies have implicated FNMTC as an independent risk factor for increased aggressiveness of thyroid cancers (27), which may explain the critical and serious condition of the proband. To the best of our knowledge, no reports have studied FNMTC in UTC patients. It is, therefore, imperative to perform thyroid ultrasound testing on family members of patients diagnosed with UTC.

Overall, this report describes the genetic characteristics of a UTC patient, with distant metastases, and the obvious benefits of targeted therapy using anlotinib. We illustrate that a combination of driver mutations, detected by NGS, could directly guide understanding of the origin of tumors and the corresponding targeted therapy. Taken together, our findings indicate that this treatment could be an acceptable option for the urgent management of UTC.

## Data Availability Statement

The raw data supporting the conclusions of this article will be made available by the authors, without undue reservation.

## Ethics Statement

Written informed consent was obtained from the patient’s relative for the publication of any potentially identifiable images or data included in this article.

## Author Contributions

XL, QL, XZ, DC, LC, ZD, and JH collected this case. SZ and JL provided pathology information of this case. HZ and DW provided bioinformatics analysis of NGS data. QL and XZ wrote the manuscript. JF, YF, SW, TM, and XL modified this paper. All authors contributed to the article and approved the submitted version.

## Funding

This work was supported by grants from the Wenzhou Municipal Science and Technology Bureau of China (Y2020063 and Y20180175).

## Conflict of Interest

Authors XZ, JH, YF, HZ, DW, SW, and TM were employed by the company Genetron Health (Beijing) Technology, Co. Ltd., Beijing, China.

The remaining authors declare that the research was conducted in the absence of any commercial or financial relationships that could be construed as a potential conflict of interest.

## Supplementary Material

Supplementary Figure 1 The mutation IGV results of the patient. **(A–C)** IGV results of TERT upstream promoter mutation (c.C228T (*Chr5: 1295228*)), CCDC6-RET fusion (CCDC6{NM_005436.4}:r.1_303+1_RET{NM_020975.4}:r.2137_5617) and TP53 mutation (c.842A>T (*Chr17: 7577096*)) of the patient’s tumor sample and their corresponding IGV results of negative control sample.

Supplementary Figure 2 Immunochemistry results of each son’s tumor tissue. From top to bottom, the immunochemistry results of the eldest son, second son and youngest son’s tumor were shown. The three sons’ tumors showed negative of Napsin A, showing as “(-)”in top right corner of the graphs. Positive of TTF-1, PAX8 and TG were identified in all of the three sons’ tumors, presenting as “(+)”in top right corner of the graphs. Negative results from negative control samples were also shown. The immunohistochemical tests of the patient and her three sons were repeated simultaneously, so the same negative controls were used. Scale bar was in the lower right corner of each image. All images were magnified by 200 times.

Supplementary Figure 3 IGV results of BRAF mutation (c.1799T>A *(Chr7: 140453136)*) of the tumor samples of the patient’s three sons. Corresponding IGV results of negative control sample were also provided.
