# Supplementary material for: Case Report: Next-Generation Sequencing Reveals Tumor Origin in a Female Patient With Brain Metastases
Source: Front Oncol. 2021 Apr 12;11:569429. doi: 10.3389/fonc.2021.569429 (PMC8072118; doi:10.3389/fonc.2021.569429)
Supplement: Supplementary Figure 1 — The mutation IGV results of the patient. (A–C) IGV results of TERT upstream promoter mutation (c.C228T (Chr5: 1295228)), CCDC6-RET fusion (CCDC6{NM_005436.4}:r.1_303+1_RET{NM_020975.4}:r.2137_5617) and TP53 mutation (c.842A>T (Chr17: 7577096)) of the patient’s tumor sample and their corresponding IGV results of negative control sample. [file Presentation_1.pptx]

## Slide 1
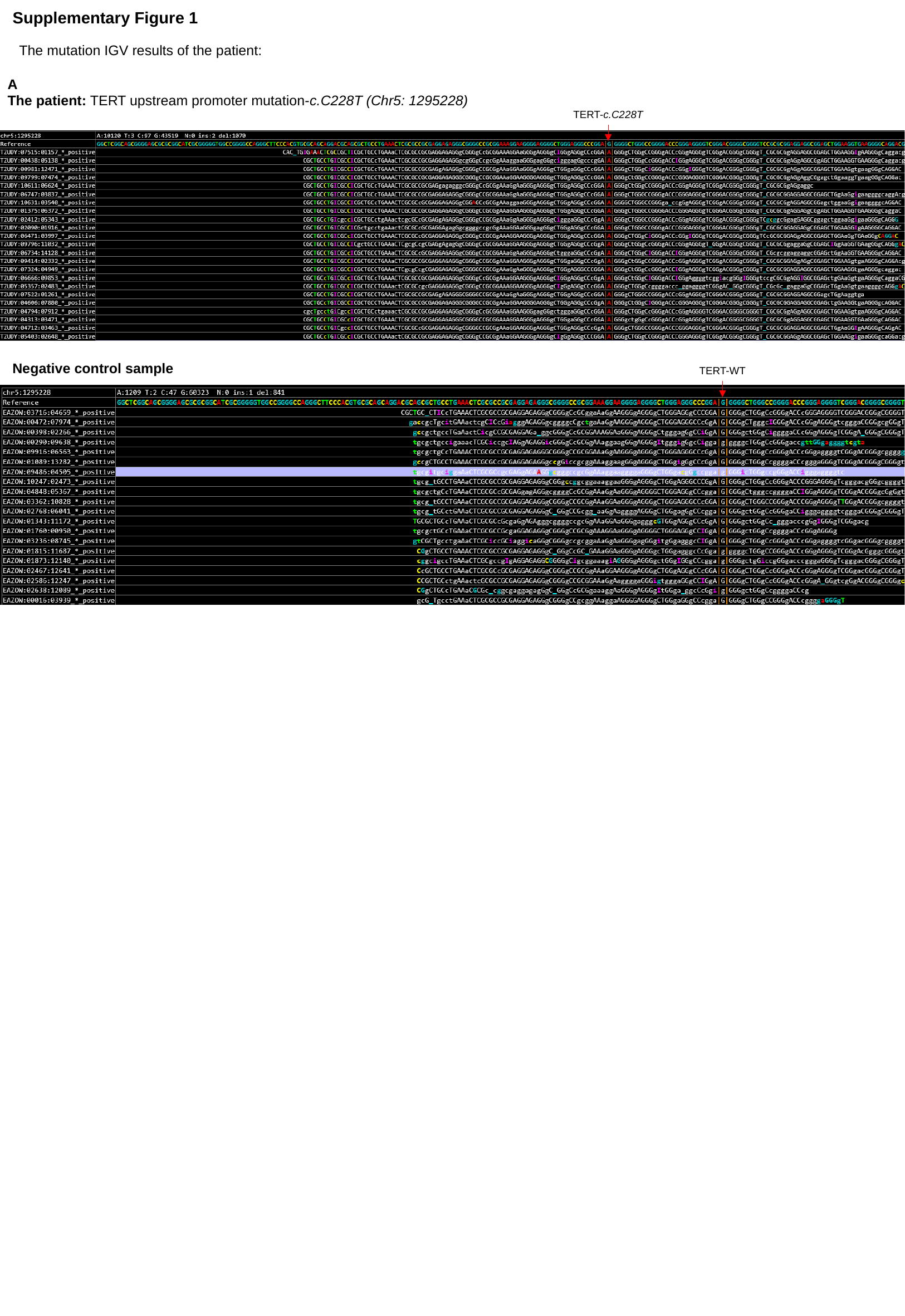

Supplementary Figure 1
The mutation IGV results of the patient:
A
The patient: TERT upstream promoter mutation-c.C228T (Chr5: 1295228)
TERT-c.C228T
Negative control sample
TERT-WT

## Slide 2
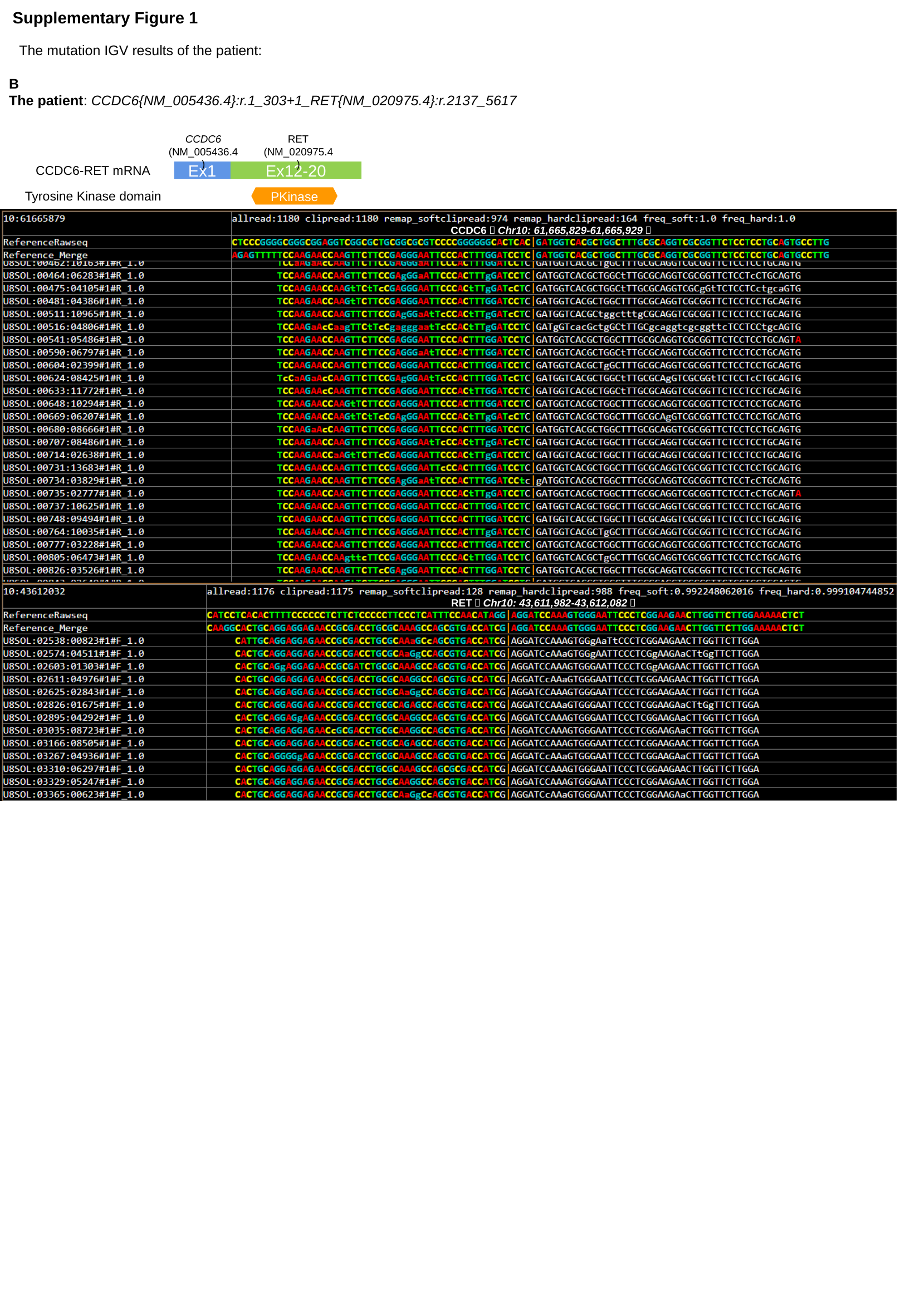

Supplementary Figure 1
The mutation IGV results of the patient:
B
The patient: CCDC6{NM_005436.4}:r.1_303+1_RET{NM_020975.4}:r.2137_5617
CCDC6
(NM_005436.4)
RET
(NM_020975.4)
CCDC6-RET mRNA
Ex1
Ex12-20
Tyrosine Kinase domain
PKinase
CCDC6（Chr10: 61,665,829-61,665,929）
RET（Chr10: 43,611,982-43,612,082）
Negative control sample

## Slide 3
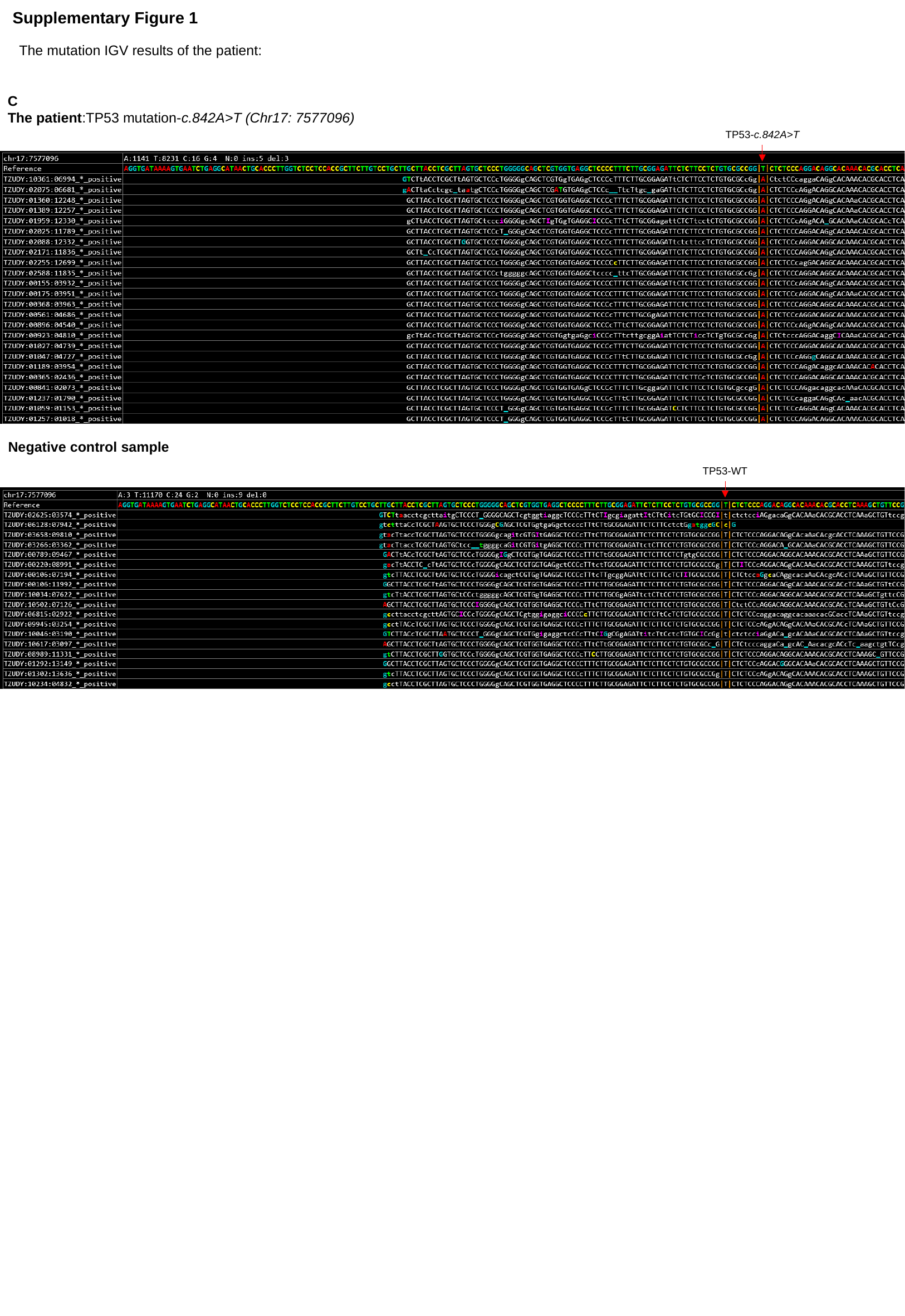

Supplementary Figure 1
The mutation IGV results of the patient:
C
The patient:TP53 mutation-c.842A>T (Chr17: 7577096)
TP53-c.842A>T
Negative control sample
TP53-WT

## Slide 4
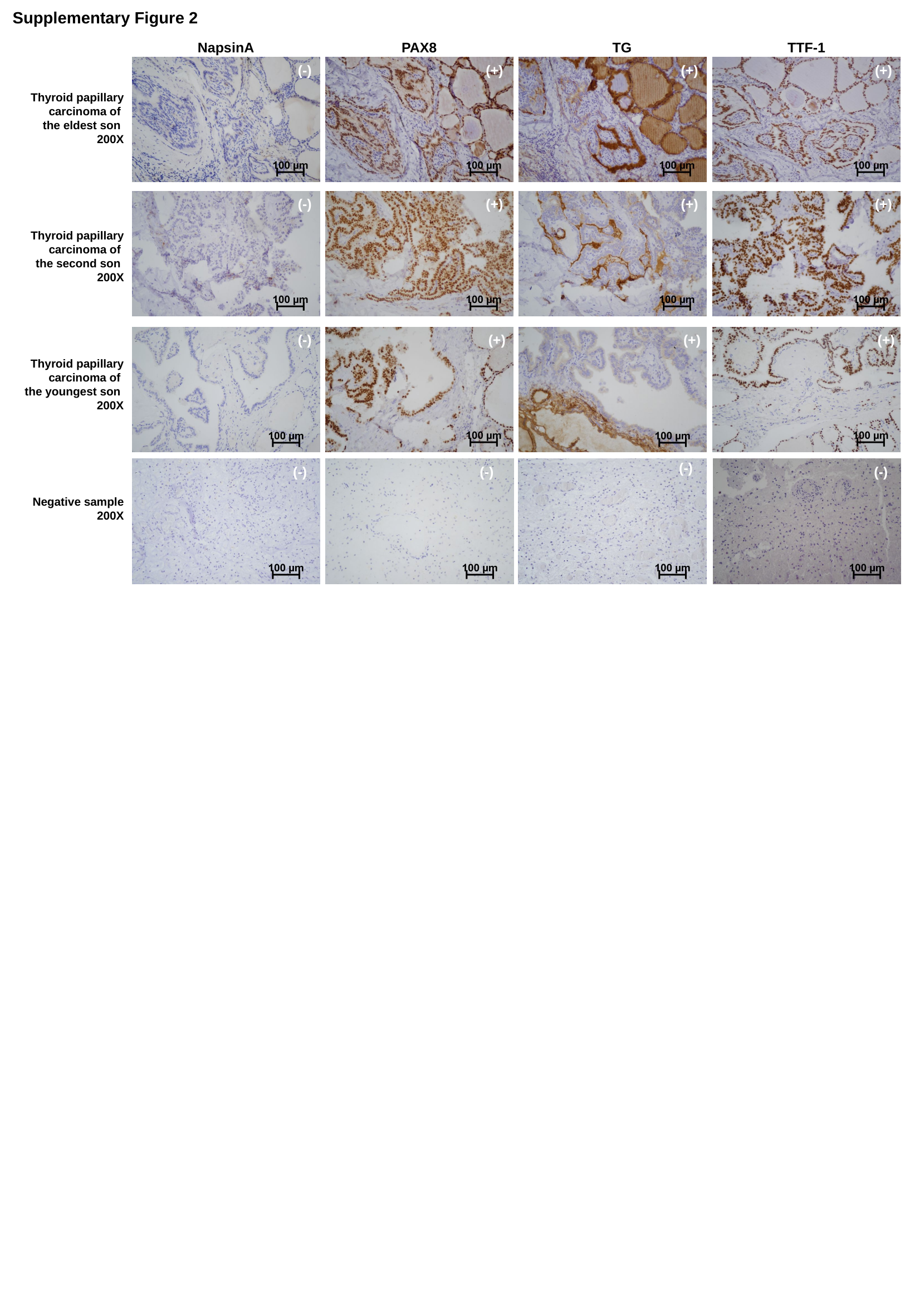

Supplementary Figure 2
NapsinA
PAX8
TG
TTF-1
(-)
(+)
(+)
(+)
Thyroid papillary carcinoma of
the eldest son
200X
(-)
(+)
(+)
(+)
Thyroid papillary carcinoma of
the second son
200X
(-)
(+)
(+)
(+)
Thyroid papillary carcinoma of
the youngest son
200X
(-)
(-)
(-)
(-)
Negative sample
200X

## Slide 5
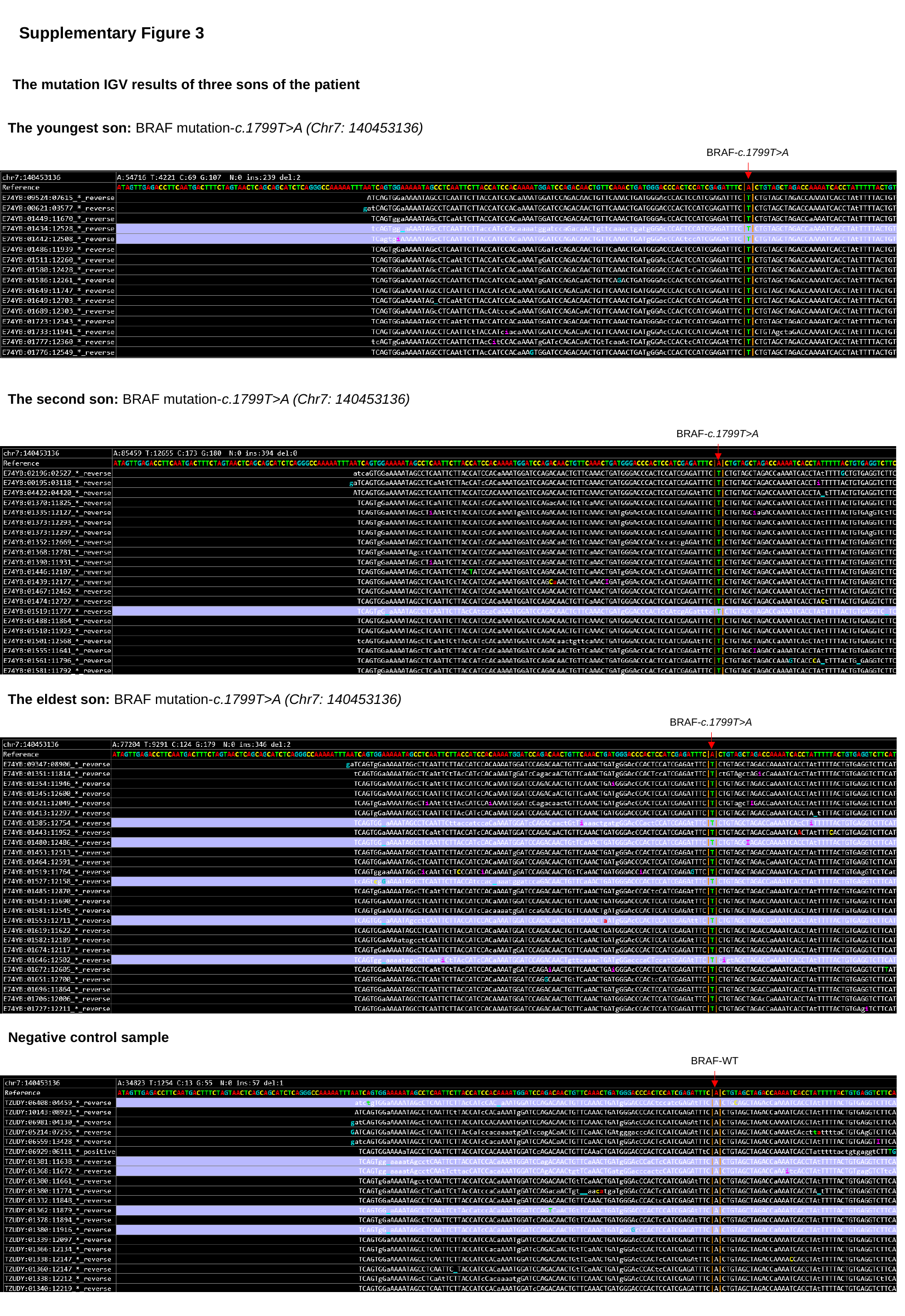

Supplementary Figure 3
The mutation IGV results of three sons of the patient
The youngest son: BRAF mutation-c.1799T>A (Chr7: 140453136)
BRAF-c.1799T>A
The second son: BRAF mutation-c.1799T>A (Chr7: 140453136)
BRAF-c.1799T>A
The eldest son: BRAF mutation-c.1799T>A (Chr7: 140453136)
BRAF-c.1799T>A
Negative control sample
BRAF-WT
