# Supplementary material for: Case Report: Next-Generation Sequencing Reveals Tumor Origin in a Female Patient With Brain Metastases
Source: Front Oncol. 2021 Apr 12;11:569429. doi: 10.3389/fonc.2021.569429 (PMC8072118; doi:10.3389/fonc.2021.569429)
Supplement: Supplementary file 2 [file DataSheet_1.docx]

**Material and methods**

**Details on the immunohistochemistry (IHC) staining of NapsinA, TG, TTF-1 and PAX8**

At our center, IHC staining of NapsinA, TG, TTF-1 and PAX8 were performed on paraffin embedded tumor tissue sections and negative control samples using a Ventana BenchMark XT automated slide-processing system (Ventana Medical Systems, Basel, Switzerland), and using commercially available antibodies, as follows: napsin A (mouse monoclonal, clone MX015, Item No. MAB-0704, MXB, FuZhou), TTF-1 (mouse monoclonal, clone SPT24, Item No. MAB-0599, MXB, FuZhou), Thyroglobulin (TG) (rabbit monoclonal, clone OTI8F2, Item No. ZM-0241, ZSGB-BIO, Beijing), and PAX-8 (rabbit monoclonal, clone GR002, Item No. GT210207, GeneTech, Shanghai). For Napsin A, granular cytoplasmic staining of tumor cells was classified as positive, and the absence of strong granular cytoplasmic staining was classified as negative. For TTF-1 and PAX-8, strong nuclear staining of tumor cells was classified as positive, while cytoplasmic staining for TG were classified as positive. More than 1% expression of these markers within tumor cells observed was considered positive.

**Details on the genetic analysis**

The molecular analysis was similar as previous report (1). Briefly, DNA and total RNA were extracted from resected tumor samples and negative control samples using the AllPrep DNA/RNA Mini Kit (QIAGEN), and their concentrations determined using a Qubit 3.0 Fluorometer（Thermo Fisher Scientific). NGS libraries, which were started with 10 ng of DNA and 10 ng of RNA, were prepared by the FSZ-Thyroid NGS Panel V1, a panel for the detection of more 1000 hotspots of 16 thyroid cancer-related genes (*AKT1, BRAF, CTNNB1, EIF1AX, EZH1, GNAS, HRAS, KRAS, NRAS, PIK3CA, RET, SPOP, TERT, TP53, TSHR, and ZNF148*) and 46 types of gene fusions occurring in thyroid cancer (*RET, PPARG, NTRK1, NTRK3, BRAF, ALK, THADA*, and others in RNA). After normalization on the Ion Chef, the libraries were then sequenced on the Ion Proton (Thermo Fisher Scientific) platform. Data analysis were performed using the Torrent Suite (version 5.2.2; Thermo Fisher Scientific).

**Reference**

1. Song Y, Xu G, Ma T, Zhu Y, Yu H, Yu W, Wei W, Wang T, Zhang B. Utility of a multigene testing for preoperative evaluation of indeterminate thyroid nodules: A prospective blinded single center study in China. Cancer Med (2020) 9:8397–8405. doi:10.1002/cam4.3450
